# Supplementary material for: Ocimum Species: A Review on Chemical Constituents and Antibacterial Activity
Source: Molecules. 2022 Sep 26;27(19):6350. doi: 10.3390/molecules27196350 (PMC9573401; doi:10.3390/molecules27196350)
Supplement: Supplementary file 1 [file molecules-27-06350-s001.zip › molecules-1921454-supplementary.pdf]

Supplementary Materials

Ocimum species: A Review on Chemical Constituents and Antibacterial Activity

Hendra Dian Adhita Dharsono [1,*], Salsabila Aqila Putri [2], Dikdik Kurnia [2], Dudi Dudi [3] and Mieke Hemiawati Satari [4]

[1] Department of Conservative Dentistry, Faculty of Dentistry, Universitas Padjadjaran, Sumedang 45363, West Java, Indonesia
[2] Department of Chemistry, Faculty of Mathematics and Natural Sciences, Universitas Padjadjaran, Sumedang 45363, West Java, Indonesia
[3] Department of Livestock Production, Faculty of Animal Husbandry, Universitas Padjadjaran; Sumedang 45363, West Java, Indonesia
[4] Department of Oral Biology, Faculty of Dentistry, Universitas Padjadjaran, Sumedang 45363, West Java, Indonesia
* Correspondence: adhita.dharsono@fkg.unpad.ac.id; Tel.: +628156223343

2. Chemical Constituents and Antibacterial Activity of *Ocimum* Species

2.1. Ocimum americanum

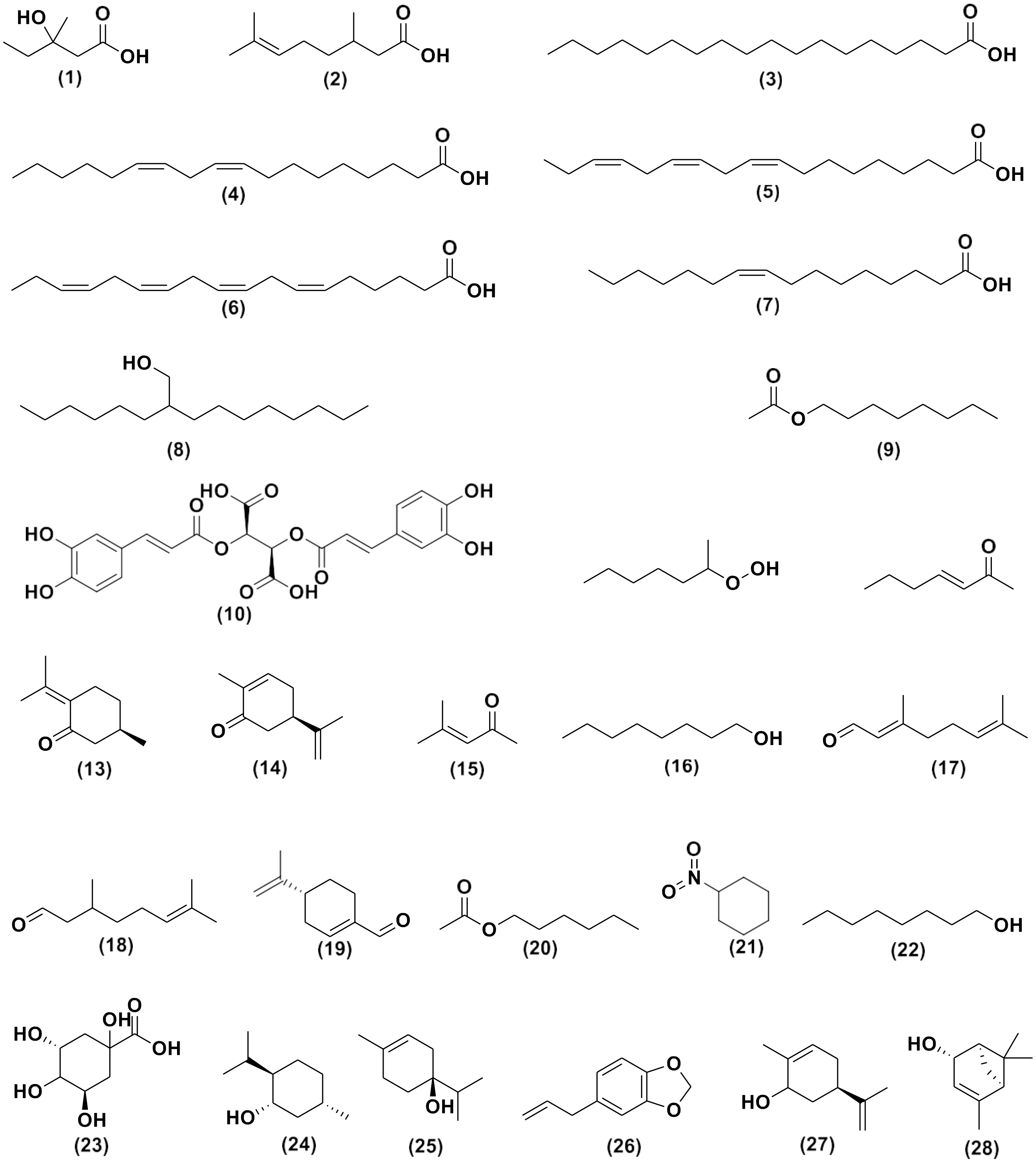

Figure S1. *Cont.*

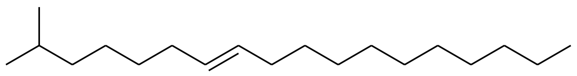
(29)

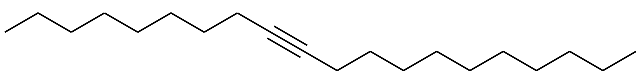
(30)

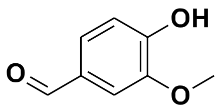
(31)

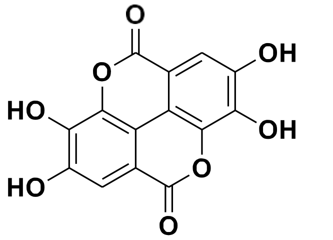
(32)

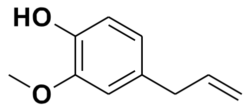
(33)

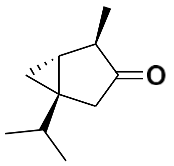
(34)

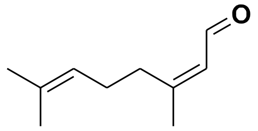
(35)

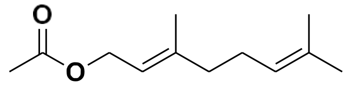
(36)

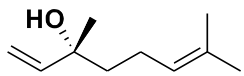
(37)

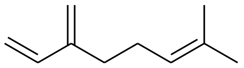
38

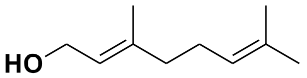
(39)

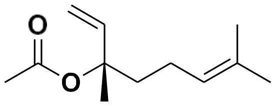
(40)

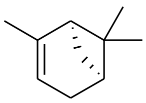
(41)

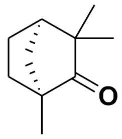
(42)

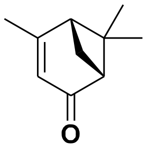
(43)

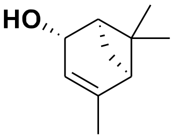
(44)

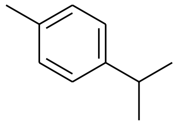
(45)

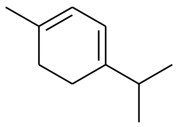
(46)

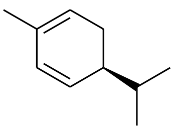
(47)

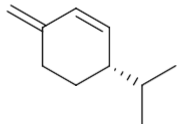
(48)

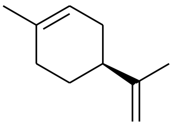
(49)

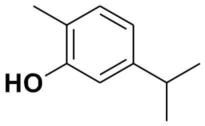
(50)

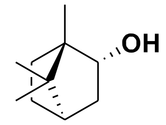
(51)

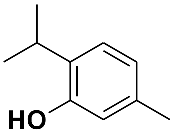
(52)

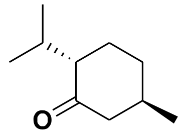
(53)

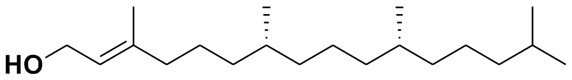
(54)

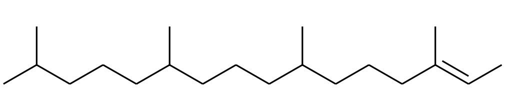
(55)

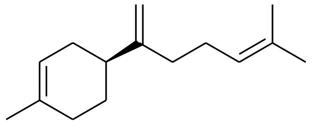
(56)

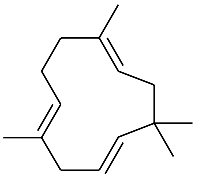
(57)

Figure S1. *Cont.*

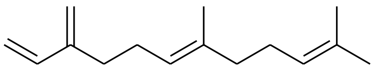
(58)

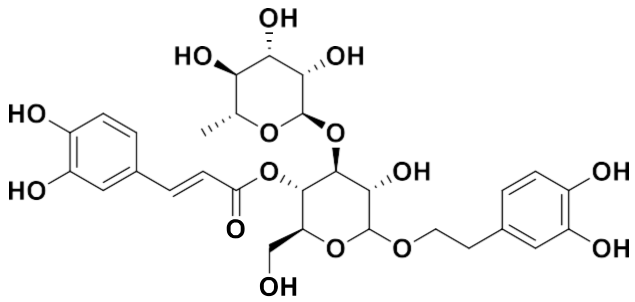
(59)

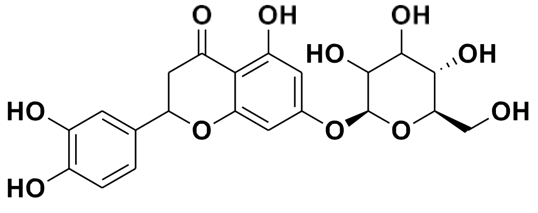
(63)

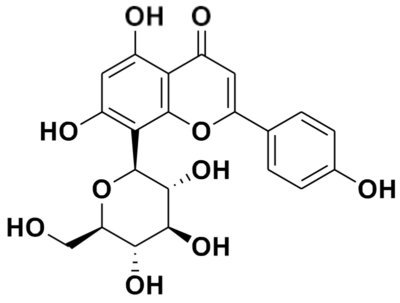
(64)

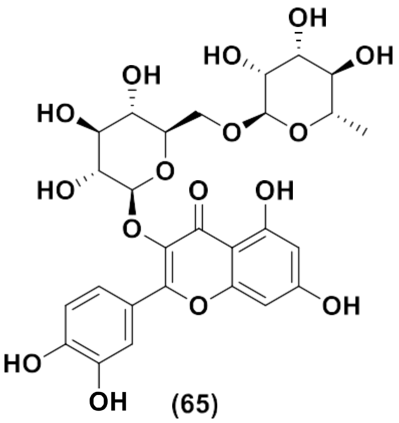
(65)

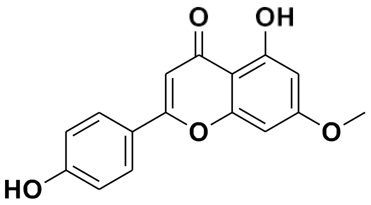
(66)

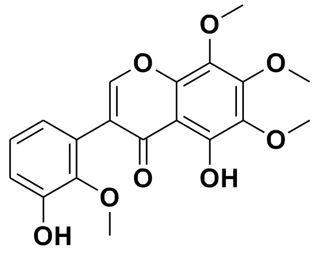
(67)

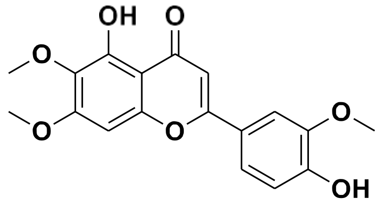
(68)

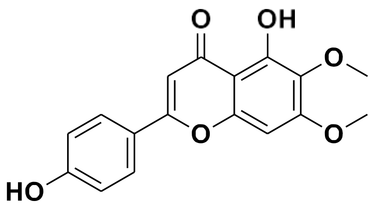
(69)

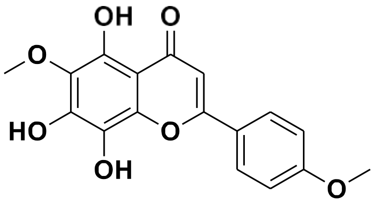
(70)

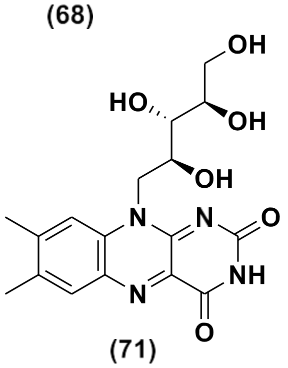
(71)

Figure S1. Chemical Compound Structures of *O. americanum*

2.2. Ocimum basilicum

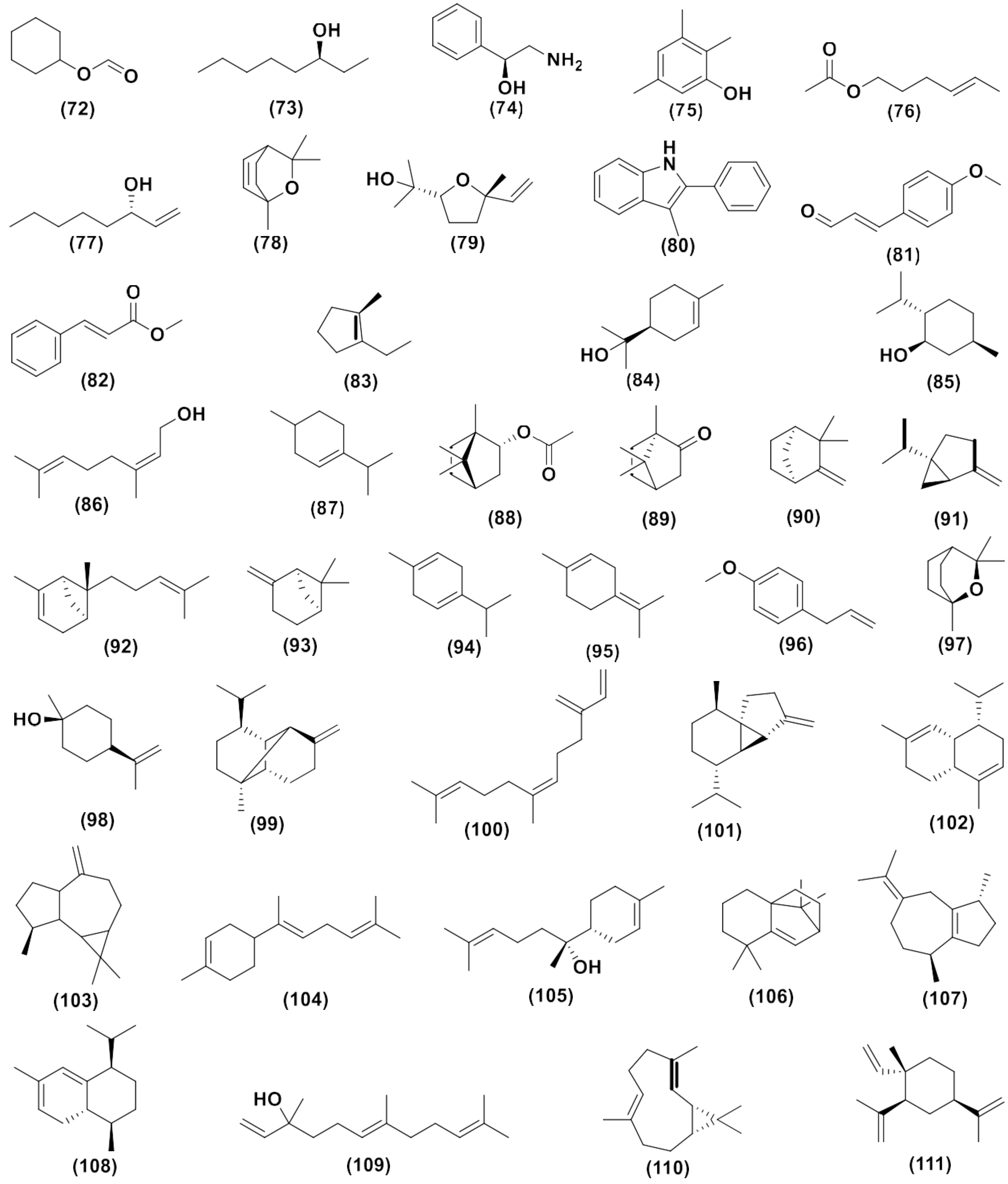

Figure S2. *Cont.*

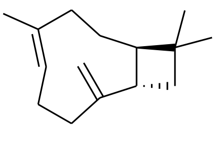
(112)
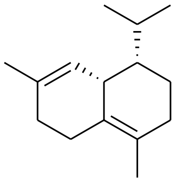
(113)
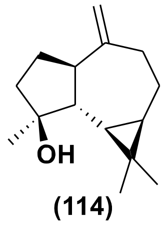
(114)
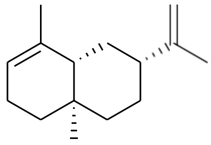
(115)
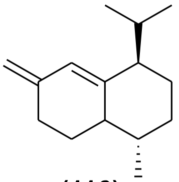
(116)
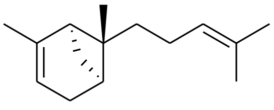
(117)
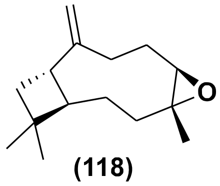
(118)
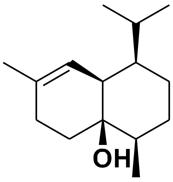
(119)

Figure S2. Chemical Compound Structures of *O. basilicum*

2.3. *Ocimum gratissimum*

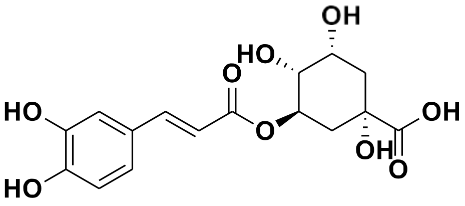
(120)
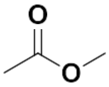
(121)
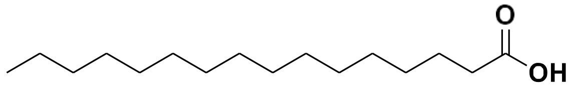
(122)
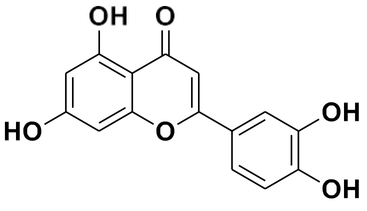
(123)
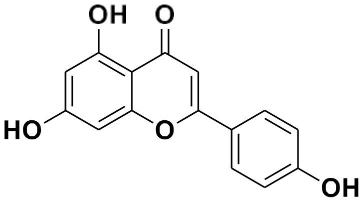
(124)
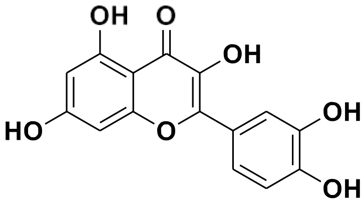
(125)
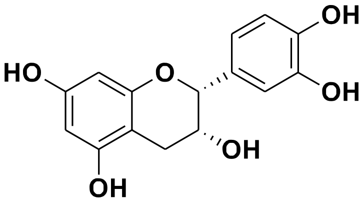
(126)
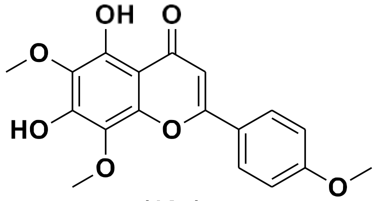
(127)
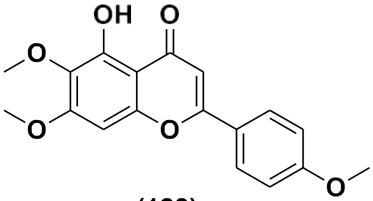
(128)
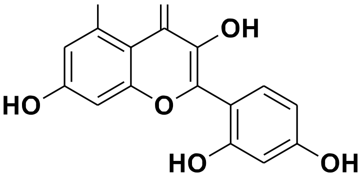
(129)
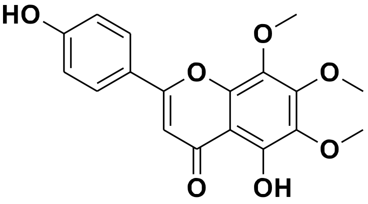
(130)
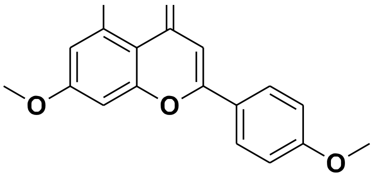
(131)

Figure S3. *Cont.*

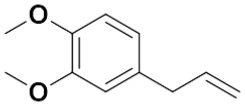
(132)

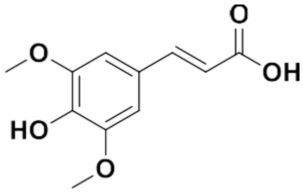
(133)

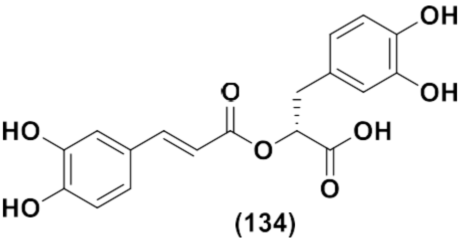
(134)

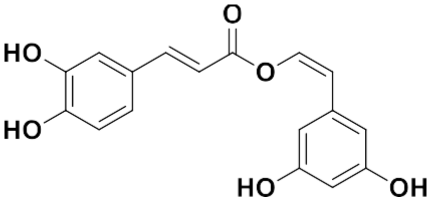
(135)

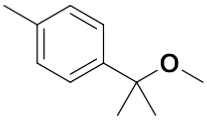
(136)

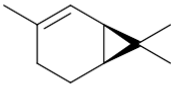
(137)

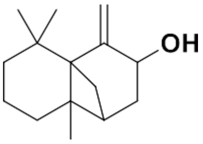
(138)

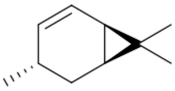
(139)

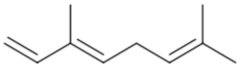
(140)

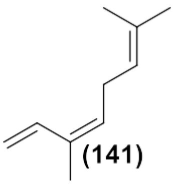
(141)

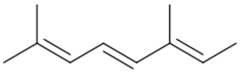
(142)

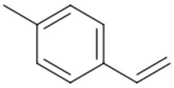
(143)

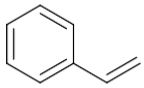
(144)

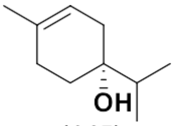
(145)

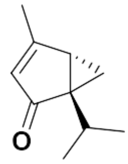
(146)

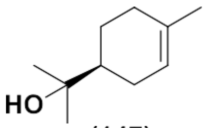
(147)

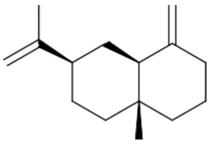
(148)

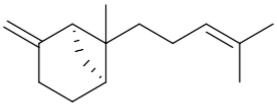
(149)

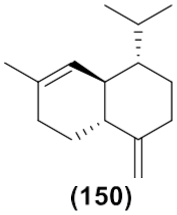
(150)

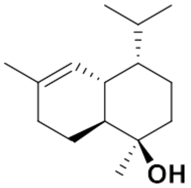
(151)

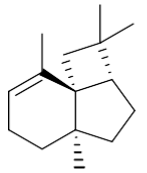
(152)

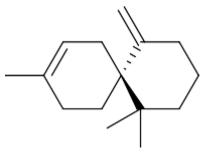
(153)

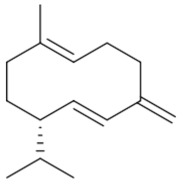
(154)

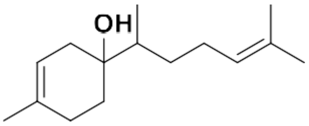
(155)

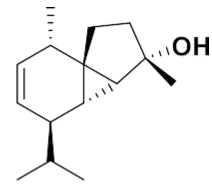
(156)

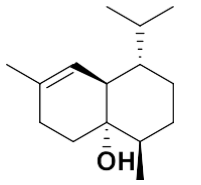
(157)

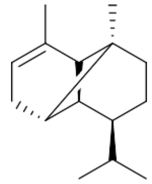
(158)

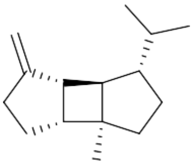
(159)

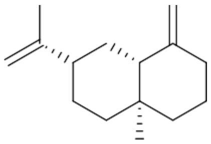
(160)

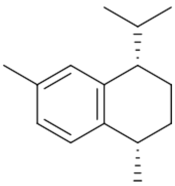
(161)

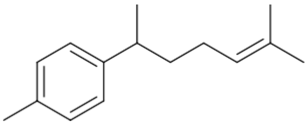
(162)

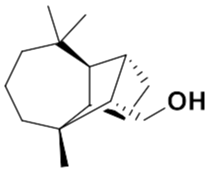
(163)

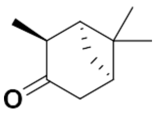
(164)

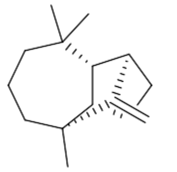
(165)

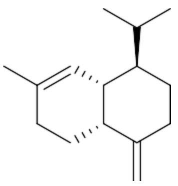
(166)

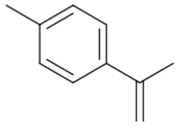
(167)

Figure S3. *Cont.*

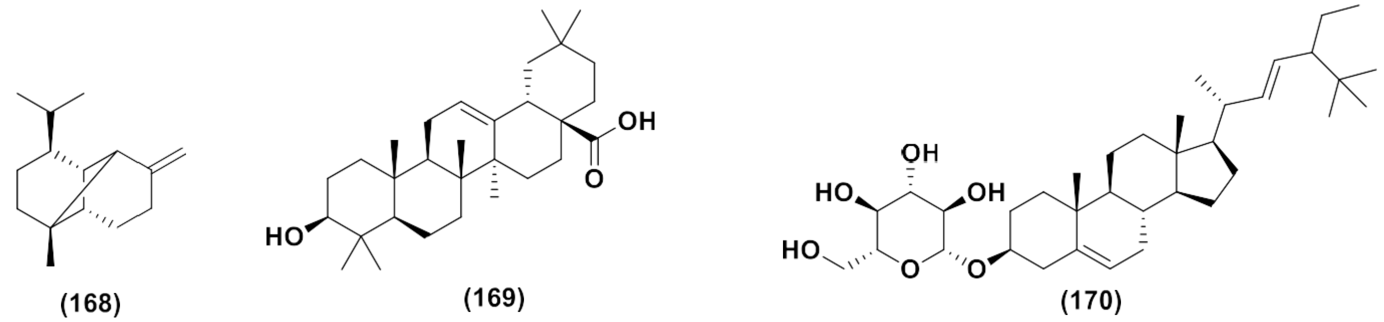

Figure S3. Chemical Compound Structures of *O. gratissimum*

2.4. *Ocimum campechianum*

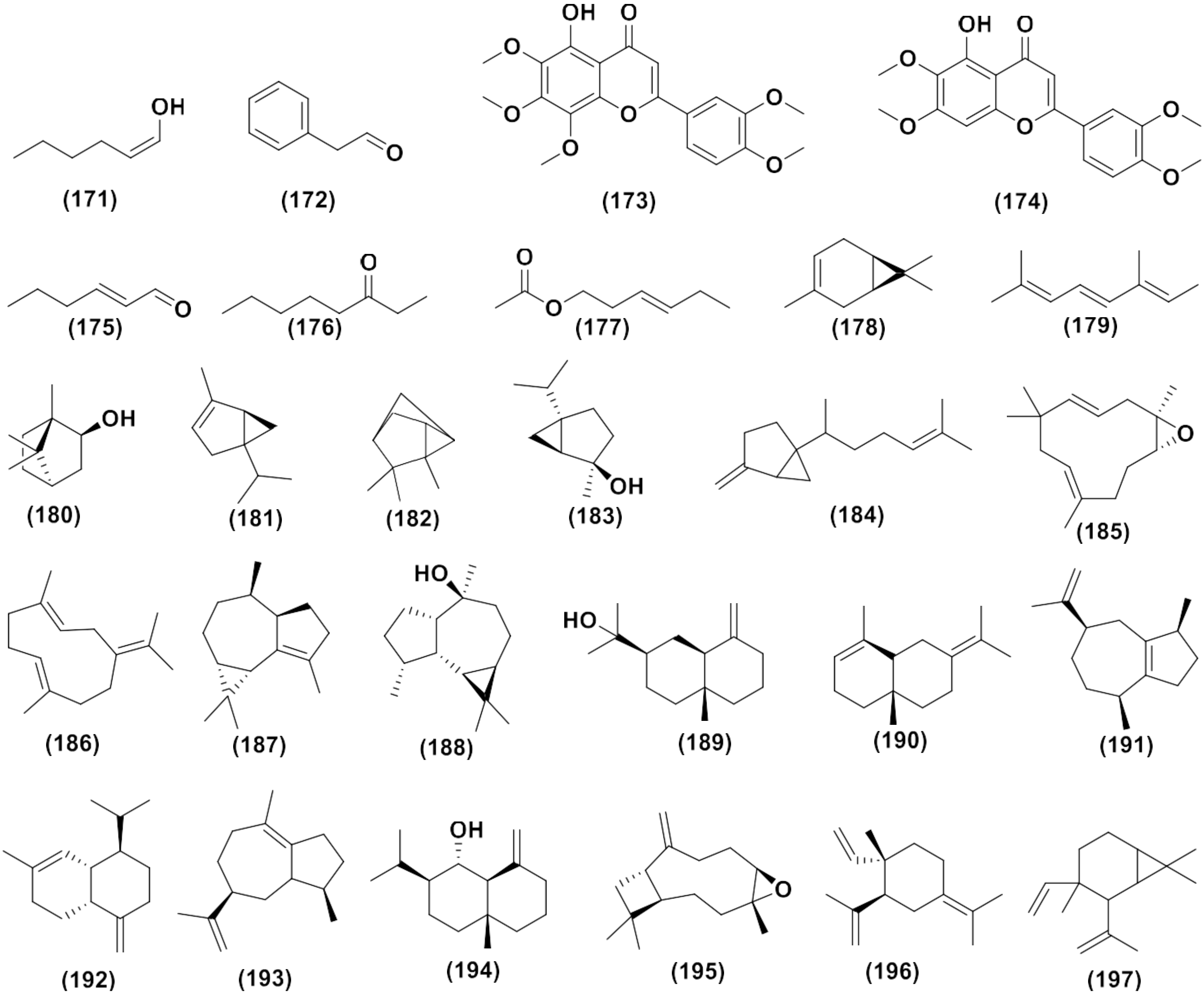

Figure S4. Chemical Compound Structures of *O. campechianum*

2.5. Ocimum sanctum

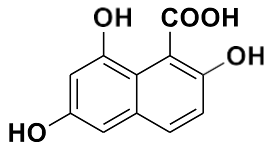
(198)

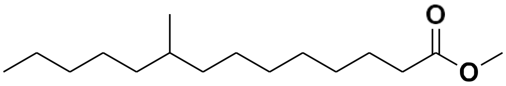
(199)

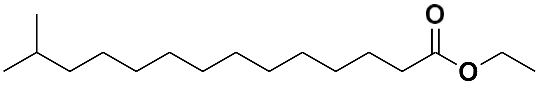
(200)

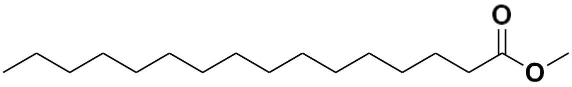
(201)

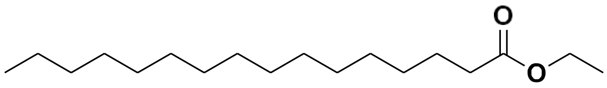
(202)

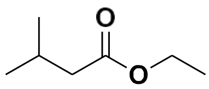
(203)

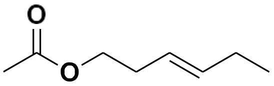
(204)

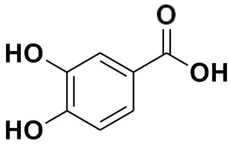
(205)

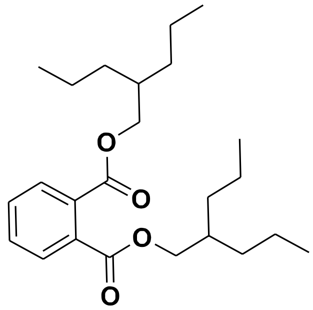
(206)

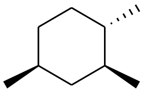
(207)

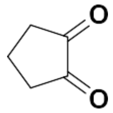
(208)

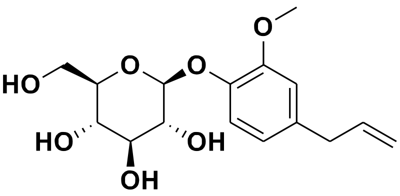
(209)

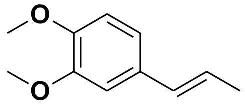
(210)

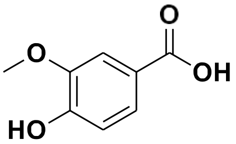
(211)

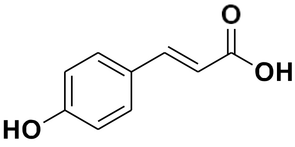
(212)

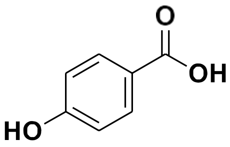
(213)

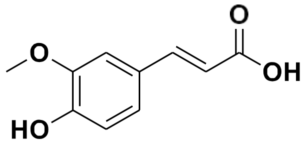
(214)

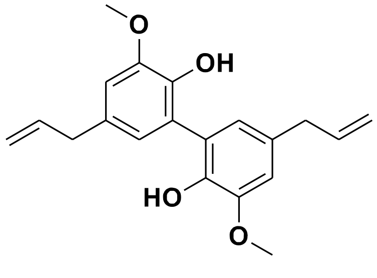
(215)

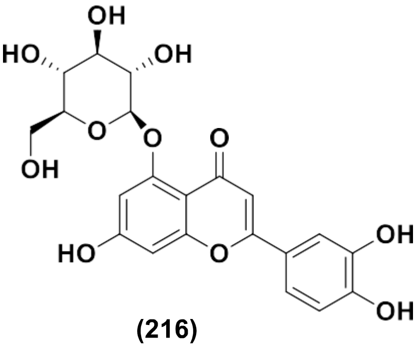
(216)

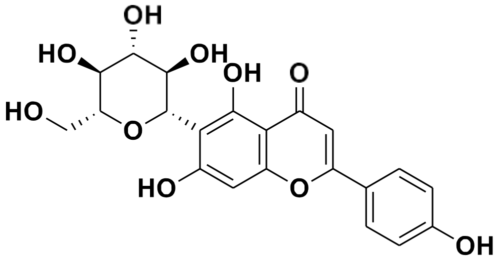
(217)

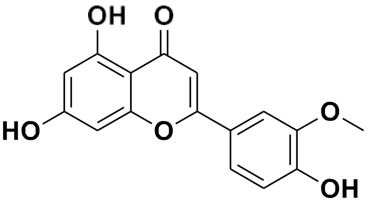
(218)

Figure S5. *Cont.*

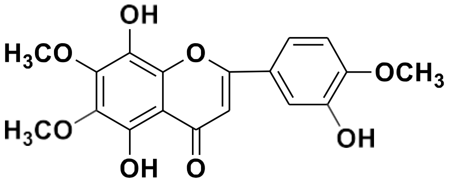
(219)

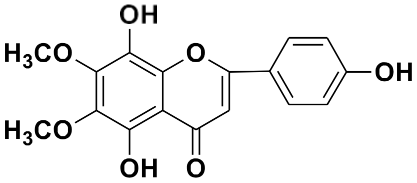
(220)

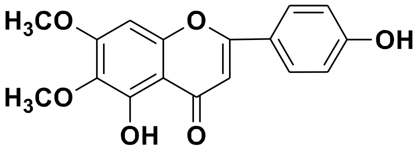
(221)

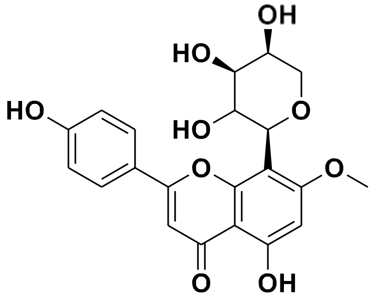
(222)

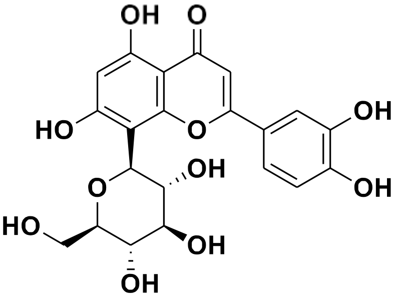
(223)

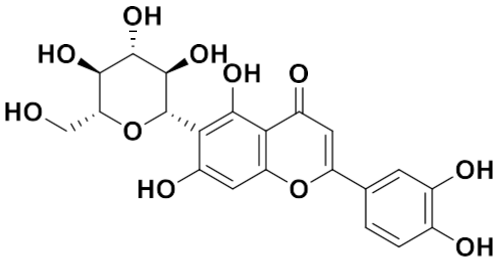
(224)

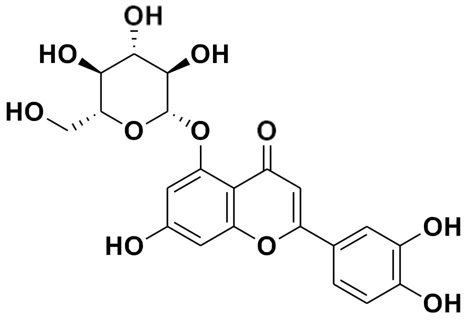
(225)

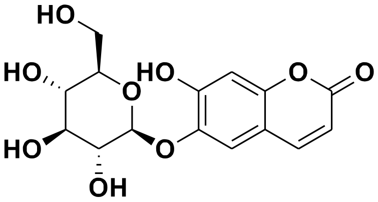
(226)

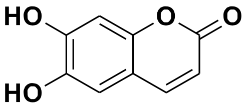
(227)

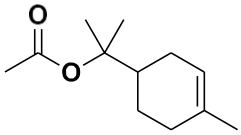
(228)

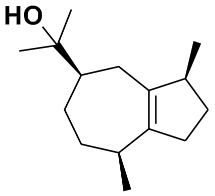
(229)

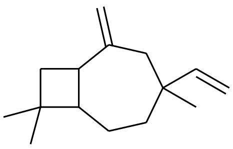
(230)

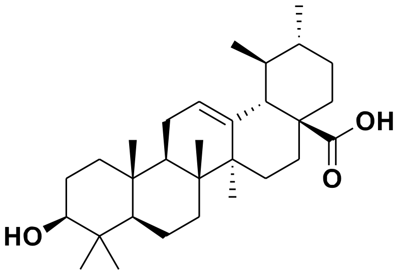
(231)

Figure S5. Chemical Compound Structures of *O. sanctum*
